# Healthcare resource utilization in the management of hypophosphatasia in three patients displaying a spectrum of manifestations

**DOI:** 10.1186/s13023-018-0869-4

**Published:** 2018-08-16

**Authors:** Anjali B. Daniel, Vrinda Saraff, Nick J. Shaw, Robert Yates, M. Zulf Mughal, Raja Padidela

**Affiliations:** 1 Department of Paediatric Endocrinology & Metabolic Bone Diseases, Royal Manchester Children’s Hospital, Oxford Road, Manchester, M13 9WL UK; 2 Department of Endocrinology and Diabetes, Birmingham Women’s and Children’s Hospital, Steelhouse Lane, Birmingham, B4 6NH UK; 3 Institute of Metabolism and Systems Research, University of Birmingham, Edgbaston, Birmingham, B15 2TT UK; 4 Department of Paediatric Intensive Care Unit, Royal Manchester Children’s Hospital, Oxford Road, Manchester, M13 9WL UK; 5 Faculty of Biology, Medicine and Health, University of Manchester, Manchester Academic Health Science Centre, Manchester, UK

**Keywords:** Hypophosphatasia, Enzyme replacement therapy, Hospitalization, Ambulatory care, Health services research, Case report, Healthcare utilization

## Abstract

**Background:**

Hypophosphatasia (HPP) is a rare, heterogeneous disease caused by low tissue-nonspecific alkaline phosphatase activity and associated with a range of signs and symptoms, including bone mineralization defects, respiratory problems, seizures, premature tooth loss, and fractures. Data from patients with HPP and their healthcare resource utilization are lacking. We evaluated healthcare utilization for 3 patients with differing severities of HPP.

**Results:**

Patient 1 had perinatal HPP (received enzyme replacement therapy asfotase alfa under a compassionate use program), Patient 2 had infantile HPP, and Patient 3 had childhood HPP. Healthcare resources used in the National Health Service, England, were identified from coded activities in the hospital database and detailed medical records. These data showed that healthcare utilization was directly related to disease severity. Patient 1 had respiratory complications necessitating prolonged admission for ventilation from birth. Over 2.5 years, this patient was hospitalized 725 days, with visits from 16 specialists. Patient 2 had HPP-associated signs and symptoms starting in infancy, was treated for craniosynostosis, experienced multiple fractures, and required outpatient management for > 18 years. Patient 3 developed signs and symptoms of HPP in childhood and received outpatient and day case treatment for dental, orthopedic, and cardiovascular problems over 24 years. Healthcare utilization varied with severity and complexity of disease manifestations between these patients.

**Conclusions:**

With the recent approval of asfotase alfa for HPP, data from this analysis may help mobilize multidisciplinary healthcare resources for management of HPP by elucidating healthcare resource needs of patients who show a spectrum of clinical manifestations of HPP.

## Background

Hypophosphatasia (HPP) is a rare, inherited, systemic, metabolic disease caused by low tissue-nonspecific alkaline phosphatase (TNSALP) activity [1–5], which results in accumulation of the TNSALP substrates inorganic pyrophosphate (PPi) and pyridoxal 5′-phosphate (PLP) [6]. In HPP, low TNSALP activity results in extracellular accumulation of PPi, blocking hydroxyapatite crystal growth and inhibiting bone mineralization [3, 7, 8]. Consequently, calcium and PPi accumulate in the bloodstream, causing disturbances in calcium/phosphate homeostasis [9]. Under normal circumstances, TNSALP dephosphorylates PLP, the circulating form of vitamin B_6_, producing pyridoxal. Pyridoxal crosses the blood-brain barrier and is rephosphorylated into PLP, where it plays an important role in the synthesis of multiple neurotransmitters. A deficiency of PLP in the central nervous system in HPP can result in seizures [10, 11].

Because HPP is rare, its true incidence and prevalence remain unknown [3]. The incidence of HPP in Toronto, based on local birth rates for Ontario, Canada, in 1957, was estimated at 1:100,000 [4]. Based on molecular diagnosis of 65 French cases from 2000 to 2009, the prevalence of the more severe perinatal and infantile HPP was estimated at 1:300,000 in Europe [5]. However, estimates based on penetrance suggested that the prevalence of moderate HPP due to heterozygous mutations may be much higher, at 1:6370 births [5].

HPP is a heterogeneous disease, with skeletal signs that can manifest in utero through adulthood [12–18]. Low TNSALP activity exerts effects on multiple systems, including the respiratory [10, 19, 20], central nervous [10, 13, 21, 22], renal [10, 23, 24], musculoskeletal [10, 16, 21, 25], and immune systems [14, 26], and dental structures [14, 27, 28].

For perinatal and infantile HPP patients who have life-threatening disease, such as those with respiratory failure secondary to hypomineralization of the chest, the 5-year mortality rate is 73% [29]. Older children and adults with HPP may experience high fracture rates, orthopedic and dental signs and symptoms, pain, impaired mobility, need for assistive walking devices, decreased functional status, and impairments in activities of daily living [14, 15, 30]. These findings are supported by results from 2 patient surveys, the Hypophosphatasia Impact Patient Survey and the Hypophosphatasia Outcomes Study Telephone Interview, which showed that HPP is associated with a high burden of disease and that substantial morbidity can develop during the lifetime of a patient with HPP, regardless of the age of presentation of signs and symptoms of HPP [30].

Although rare diseases by definition affect a small proportion of the overall population, they can have a major impact on the healthcare system [31]. Some studies have attempted to calculate the economic burden and health-related quality of life in patients with other rare, debilitating diseases, such as Duchenne muscular dystrophy and cystic fibrosis, with results showing a considerable burden on both patients and society [32–34]. To the best of our knowledge, healthcare resource utilization for different onsets of HPP has not been previously described.

Given the heterogeneous nature of the presenting signs and symptoms of HPP, healthcare resource utilization could vary considerably. For example, patients with severe hypomineralization of the chest with secondary respiratory complications are likely to require ventilatory support [3] and intensive care management compared with those with mild to moderate signs, symptoms, and complications of their disease.

To better understand the healthcare resource utilization of patients with differing manifestations of HPP, we reviewed 3 representative cases within the UK National Health Service network, including 1 patient with perinatal HPP who received treatment with asfotase alfa, a human recombinant TNSALP enzyme replacement therapy (Strensiq®; Alexion Pharmaceuticals, Inc., New Haven, CT) that is approved for the treatment of HPP in several countries [35–39]. The 2 other patients, who had infantile and childhood HPP, were treated before asfotase alfa was available.

## Methods

### Study design

We reviewed the charts of 3 patients who represented a spectrum of severity of HPP and had sufficient follow-up data available to assess healthcare resource utilization. The review included all available data starting from their first visit at the participating hospitals (Manchester University NHS Foundation Trust and Birmingham Women’s and Children’s Hospital in England). Informed consent was obtained from each patient and/or their family or guardian. Approval of the protocol was not required by an institutional review board or ethics committee for this type of study.

The 3 patients had clinical, biochemical, and genetic evidence of HPP. They were selected because they manifested the different signs, symptoms, complications, and types of HPP. Patient 1 had signs and symptoms during the perinatal period and received treatment with asfotase alfa through a compassionate use program, Patient 2 had signs and symptoms as an infant, and Patient 3 had signs and symptoms during childhood. Information collected included patient characteristics and history of progress and clinical management of HPP, such as outpatient visits with various specialists, inpatient admissions, and investigations and procedures performed.

Healthcare resources identified from medical records were recorded, grouped, and itemized according to inpatient admissions (including day case procedures, defined as planned hospital admissions lasting ≤1 day for a procedure or surgery that required use of a bed), outpatient specialist reviews, and any community support provided (e.g., long-term ventilation). Hospital admissions, along with the number and type of procedures performed, and any visits from specialist attending physicians and therapists were also recorded and itemized.

No formal statistical analyses of the data were performed. Summaries are provided by patient.

## Results

The healthcare resource utilization of 3 patients with HPP is described in detail in the patient summaries below.

### Patient summaries

#### Patient 1

A 34-week gestational age infant girl born to consanguineous parents was referred to a tertiary center for hypercalcemia and low alkaline phosphatase (ALP) activity < 20 IU/L (reference range: 75–250 IU/L). She had dysmorphic features with short limbs, craniotabes, and significant hypotonia. Her full skeletal survey revealed the characteristic features of HPP of undermineralized bones and absence of ossification in multiple bones of the skull and hand. The diagnosis of perinatal HPP was suspected and confirmed by sequencing of the *ALPL* gene; a homozygous mutation (C.1336 G > A [p.Ala446Thr]) was identified, with both parents being carriers of the mutation. Treatment with asfotase alfa enzyme replacement therapy was initiated at age 1 month through a compassionate use program.

The patient had very poor respiratory efforts, with shallow breathing requiring ventilator support from birth and prolonged intermittent positive pressure ventilation until age 12 months, when she was weaned onto continuous positive airway pressure (CPAP) ventilation. By age 16 months, she was self-ventilating for 12 h of the day. At 23 months of age, she was discharged home but required CPAP at night. At age 2.5 years, ventilatory support was completely discontinued. At age 3.5 years, she developed craniosynostosis and was awaiting craniofacial surgery at the time of this review. Other clinical challenges included poor feeding and swallowing difficulties that required nasogastric feeding from birth to age 5 months, after which she was fed through gastrostomy, with oral intake progressively improving.

##### Healthcare utilization

**Fig. 1:**
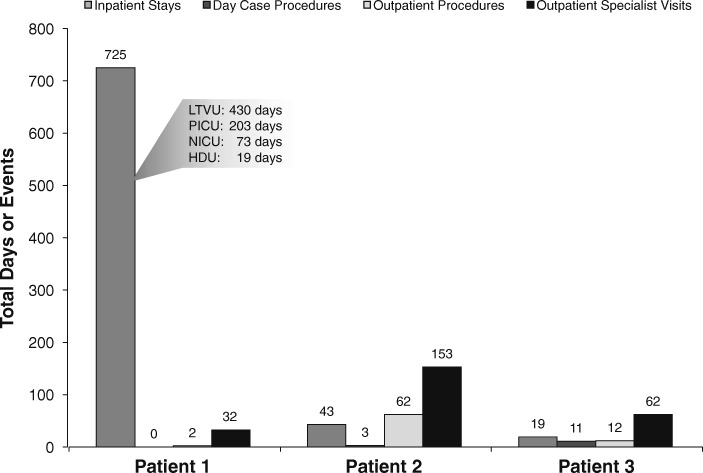
Healthcare resource utilization by patient. HDU = high-dependency unit; LTVU = long-term ventilation unit; NICU = neonatal intensive care unit; PICU = pediatric intensive care unit

Over the first 2.5 years of life, this patient was hospitalized for a total of 725 days (Table 1 and Fig. 1). Admissions associated with care in a long-term ventilation treatment unit (430 days) and a pediatric intensive care unit (PICU; 203 days) represent the majority of inpatient time. In addition, the patient was admitted to a neonatal intensive care (NICU) unit for 73 days and a high-dependency unit for 19 days.

**Table 1:** Patient 1: Resource utilization during the first 2.5 years of life

Visit type	Reason/procedure	No. of events (unless otherwise specified)
Inpatient	Inpatient stays^a^	725 days
	NICU	73 days
	Mechanical ventilation	73 days
	Chest radiographs	16
	Endotracheal intubation	3
	Ultrasonography	
	− Cranial	3
	− Abdominal	2
	− Renal tract	2
	EEG	1
	Tracheostomy	1
	Specialist visits	
	− Otorhinolaryngologist	3
	− Genetics specialist	1
	PICU	203 days
	Mechanical ventilation	203 days
	Tracheoscopy	3
	Hickman line–insertion and manipulation of line under general anesthesia	3
	Radiography	
	− Chest	3
	− Forearm	2
	Cranial CT and MRI	2
	PORT-A-CATH insertion, central line removal	1
	Neurologic procedure: intracranial pressure monitor insertion	1
	Neurologic procedure: intracranial pressure monitor removal	1
	Thoracic CT with contrast	1
	Ultrasonography	
	− Abdomen	1
	− Hips	1
	− Central veins	1
	− Doppler of neck and left leg	1
	Endotracheal intubation	1
	Specialist visits	
	− Occupational therapist, 2 times/wk	57
	− Physiotherapist, 2 times/wk	57
	− Dietician	32
	− Pediatric immunologist	12
	− Neurologist	6
	− Ophthalmologist	5
	− Speech and language specialist	5
	− Otorhinolaryngologist	4
	− Cardiologist	3
	− Neurosurgeon	2
	− Pediatric surgeon	2
	− Genetics specialist	1
	− Microbiologist	1
	− Orthopedic surgeon	1
	− Pediatric radiologist	1
	− Pulmonologist	1
	High-Dependency Unit, pediatric bed days	19 days
	Mechanical ventilation	19 days
	Renal tract ultrasonography	1
	Gastrostomy insertion	1
	Long-term Treatment Ventilation Unit	430 days
	Mechanical ventilation	430 days
	Radiography	2
	Respiratory sleep study	2
	Cranial CT	1
	Renal tract ultrasonography	1
	Gastrostomy tube insertion	1
	Specialist visits	
	− Pulmonologist	430
	− Occupational therapist, 2 times/wk	123
	− Physiotherapist, 2 times/wk	123
	− Dietician	21
	− Speech and language specialist	16
	− Neurosurgeon	4
	− Ophthalmologist	2
	− Otorhinolaryngologist	1
	− Pediatric surgeon	1
Outpatient procedure	Outpatient procedures	2
	Revision of gastrostomy tube	1
	Renal ultrasonography	1
Outpatient specialist visit	Outpatient specialist visits	32
	Pediatric endocrinologist visits (first visit and 3 follow-up attendance visits)	4
	Occupational therapist, 2 times/mo	14
	Physiotherapist, 2 times/mo	14
Community ventilation	Community ventilation	185 days
	Community long-term ventilation	185 days

*CT* computed tomography, *EEG* electroencephalogram, *MRI* magnetic resonance imaging

^a^No visits are recorded for a Pediatric Endocrinologist/Pediatrician for inpatient stays, as visits by these specialists are considered part of the standard care and typically occurred on a daily basis

The highest number of procedures was performed and the highest number of specialist visits took place while the patient was in the PICU. During these inpatient stays, the patient required visits from 16 medical specialists, with close oversight from intensivists and a pediatric endocrinologist. The pulmonologist attended to the patient across virtually all admissions (except in the NICU and high-dependency pediatric unit). Consultation for respiratory needs required 173 visits during one 425-day stay in a long-term treatment unit; besides the visits from the pediatric endocrinologist, this was the highest number of visits from any specialist across all admissions (Table 1). This patient also received physical therapy and occupational therapy sessions (57 visits each), 32 dietician visits, and 12 consultations with a pediatric immunologist (Table 1). Physical therapy and occupational therapy were key aspects of treatment of this infant to improve muscle and bone strength and prevent deformities. In the hospital, a therapist performed interventions twice a week, with each session lasting 30 to 45 min. In the outpatient setting, therapy was provided once every 2 weeks. Visits from an otorhinolaryngologist were regularly required across the NICU, PICU, and long-term treatment unit hospital admissions. Of 26 procedures performed, the most frequent was chest radiography.

Additionally, adaptive equipment was required for long-term ventilation and joint support during infancy. Components of the Symmetrikit sleep system (Symmetrikit, Bromyard Road, Ledbury, Herefordshire, UK HR8 1NS) were modified to promote proximal joint alignment in this hypotonic infant with abducted and externally rotated limbs. In addition, padded blocks were used for positioning the head to prevent skull deformities. To assess changes in gross motor, fine motor, and cognitive development, Bayley Scales of Infant and Toddler Development, Third Edition (Bayley-III), was administered every 3 months.

The majority of the admissions occurred during the first 22 months of life, after which time outpatient procedures and community-based support became the primary point of care. After discharge from hospital when the patient was age 23 months, the greatest healthcare resources need was for long-term ventilation support at home; this formed the majority of nonhospital care resource requirements and was needed for 185 days over 6.5 months (Table 1).

#### Patient 2

An 11-month-old boy was referred to a tertiary center for failure to thrive, poor muscle tone, short neck, kyphosis, and unusual spacing between teeth. He was diagnosed with infantile HPP after repeated low ALP activity test results and radiographic assessment of severe rickets-like skeletal changes and tongue-like lucencies projecting into the metaphyses. He was hospitalized multiple times for pneumonia likely related to musculoskeletal manifestation of HPP, which required treatment with intravenous antibiotics. At age 9 years, he developed persistent headaches; a magnetic resonance imaging (MRI) cranial scan confirmed craniosynostosis with Chiari malformation and cerebellar tonsillar herniation. He underwent craniovertebral decompression, with removal of the posterior arch of C1; a ventriculoperitoneal shunt was inserted to relieve intracranial pressure. He subsequently underwent 2 shunt revisions.

The patient experienced multiple fractures, starting at age 17 years, when he sustained bilateral femoral fractures when jumping off a wall; this required bilateral intramedullary rod insertion. At age 18 years, he sustained a right tibial fracture while jumping. Bone healing was delayed, but the fracture eventually healed satisfactorily. At age 20 years, he sustained bilateral femoral fractures when he rolled off his bed during a seizure and required rehabilitation for approximately 8 months.

##### Healthcare utilization

Over the first 18 years of life, the patient was hospitalized 8 times for a total of 43 days (Table 2). Of these hospitalizations, 5 separate admissions required a stay of ≥5 days; the stay for insertion of a ventriculoperitoneal shunt was 14 days.

**Table 2:** Patient 2: Resource utilization over a period of 18 years

Visit type	Reason/procedures	No. of events (unless otherwise specified)
Inpatient	Inpatient stays	43 days
	Pediatric Ward	3 days
	Renal ultrasonography	1
	Skull radiograph	1
	Cranial CT	1
	DXA	1
	Ophthalmologist assessment	1
	Pediatric Ward	3 days
	Inpatient admission for physiotherapy and occupational therapy and review by specialist	
	Neurosurgical Ward	5 days
	Craniovertebral decompression	1
	Chest radiograph	1
	Foramen magnum decompression	1
	Neurosurgical Ward	14 days
	Cranial MRI	2
	Ventriculoperitoneal shunt insertion	1
	Lumbar puncture and lumbar drain insertion	1
	Cranial CT	1
	Neurosurgical Ward	7 days
	Left ventriculoperitoneal shunt insertion, stealth guided	1
	Cranial CT	1
	Neurosurgical Ward	6 days
	Left ventriculoperitoneal shunt insertion, image guided	1
	Orthopedic Ward	5 days
	Intramedullary rod insertion for bilateral femur fractures	1
Day case	Day case procedures	3
	Dental surgery	2
	Teeth extraction	1
Outpatient procedure	Outpatient procedures	62
	Radiography	
	− Limbs	18
	− Spine	12
	− Pelvis	1
	MRI	
	− Cranial	14
	− Spine	2
	DXA	6
	Renal ultrasonography	6
	CT	
	− Cranial	2
	− Abdomen	1
Outpatient specialist visit	Outpatient specialist visits	153
	Pediatric dentist	38
	Pediatric endocrinologist (first plus follow-up attendance)	32
	Neurosurgeon	27
	General pediatrician (first plus follow-up attendance)	19
	Pediatric ophthalmologist (first plus follow-up attendance)	10
	Occupational therapist	7
	Spinal surgeon	7
	Pediatric orthopedist (first plus follow-up attendance)	5
	Pain clinics	4
	Pediatric plastic surgeon (first plus follow-up attendance)	2
	Genetic specialist	1
	Pediatric craniofacial team (first attendance)	1

*CT* computed tomography, *DXA* dual-energy x-ray absorptiometry, *MRI* magnetic resonance imaging

Outpatient specialist visits represented a significant proportion of the healthcare resource utilization by this patient (Fig. 1). Most of the outpatient specialist visits required consultation with providers in 12 specialties, including pediatric dentist (38 visits), pediatric endocrinologist (32 visits), neurosurgeon (27 visits), and general pediatrician (19 visits; Table 2). Outpatient management consisted of diagnostic imaging procedures (Table 2). The most frequent procedures were radiography of the limbs and spine, performed on 18 and 12 occasions, respectively; the patient also underwent 14 MRI cranial scans. Dental surgery and tooth extraction were performed as day case procedures for management of dental carries and malocclusion on 3 occasions.

#### Patient 3 (Some details of this case were previously described [40])

A 3-year-old girl was referred to a tertiary metabolic bone disease unit for premature loss of primary teeth with roots intact and low serum ALP activity (123 IU/L; reference range: 230–700 IU/L) [40]. Routine genetic testing revealed compound heterozygosity (c.350A > G, p.Y117C, c.400_401AC > CA, p.T134H) for different *TNSALP* missense mutations in exon 5 of the *ALPL* gene, confirming the diagnosis of HPP. On presentation, radiologic assessment of the left hand and arm showed tongue-like lucencies projecting into the metaphyses consistent with childhood HPP. She did not have any clinical features of skeletal involvement of the lower limbs and no motor developmental delay except for a mild waddling gait as a younger child. The patient had a relatively asymptomatic clinical course until she presented at age 11 years with swelling and tenderness of the left ankle that was nonresponsive to paracetamol or ibuprofen. An MRI scan of the ankle suggested a diagnosis of chronic recurrent multifocal osteomyelitis, which was subsequently confirmed by biopsy. The symptoms of pain and swelling of the lower limb joints showed spontaneous transient improvement at age 13 years. Recurring at age 14 years, the symptoms fluctuated and caused significant pain and disability. These symptoms eventually stabilized when the patient was transitioned to adult care at age 17 years. At age 18 years, she successfully underwent radiofrequency ablation for Wolff-Parkinson-White Syndrome, a cardiac disorder unrelated to HPP. The patient is now 27 years of age and has experienced an episode of metatarsal stress fracture; she also suffers from generalized aches and pain.

##### Healthcare utilization

Over 22 years, this patient was hospitalized 3 times for a total of 19 days (Table 3). Only 1 hospitalization exceeded 3 days, when the patient was admitted for 14 days to receive intravenous antibiotics for suspected osteomyelitis (Table 3).

**Table 3:** Patient 3: Resource utilization over a period of 22 years

Visit type	Reason/procedures	No. of events (unless otherwise specified)
Inpatient	Inpatient stays	19 days
	Pediatric Ward	14 days
	IV antibiotics for chronic multifocal osteomyelitis	1
	Pediatric Ward	3 days
	Bisphosphonate infusion for chronic multifocal osteomyelitis	1
	MRI scan	1
	Pediatric Ward–admission under cardiology	2 days
	Supraventricular tachycardia ablation	1
Day case	Day case procedures	11
	Restorative dentistry (elective)	3
	Radiography	
	− Skull	1
	− Left arm/hand	1
	Renal ultrasonography	1
	MRI scan of lower limbs	1
	CT scan of lower limbs	1
	Bone biopsy (elective)–admission under orthopedics	1
	Dental procedure (elective)–treatment of dental caries under local anesthesia	1
	Endoscopy and biopsy (elective)–admission under gastroenterology	1
Outpatient procedure	Outpatient procedures	12
	Radiography of limbs	9
	MRI of lower limbs with contrast	3
Outpatient specialist visit	Outpatient specialist visits	62
	Pediatric dentist	40
	Child and adolescent psychiatrist	6
	Pediatric cardiologist (first and follow-up attendance)	6
	Pediatric rheumatologist	6
	Pediatrician (first and follow-up attendance)	2
	Physiotherapist	1
	Podiatrist	1

*CT* computed tomography, *IV* intravenous, *MRI* magnetic resonance imaging

Outpatient specialist visits, outpatient procedures, and day case procedures represent the majority of healthcare resources used by this patient (Fig. 1). Seven specialists provided care for the patient; a pediatric dentist was seen on 40 occasions. Dental procedures, including restorative dentistry (performed on 3 occasions), were the most common of these. This patient was also seen by a pediatric rheumatologist and psychiatrist (Table 3).

## Discussion

The lifetime burden of HPP has a major impact on healthcare resource utilization, as well as on patients and their families. To our knowledge, this is the first evaluation of healthcare resource utilization by patients with HPP. We present 3 patients with differing clinical presentations and medical management challenges to illustrate the diversity and complexity of healthcare resource utilization for these patients in the United Kingdom.

The perinatal HPP patient (Patient 1) experienced life-threatening symptoms, required intensive care unit admission, and was the most complex to treat. As a result, this patient required the longest time in the hospital and a broader range of specialists. This example illustrates that, in addition to treatment with asfotase alfa, patients with life-threatening perinatal HPP require high-quality supportive care from a team of medical, surgical, and allied healthcare professionals. Care of such infants should ideally be provided in specialist pediatric tertiary hospitals, which can carefully coordinate a core care team of specialists. Healthcare requirements that were not taken into consideration during the evaluation of Patient 1 are the resources needed upon prenatal diagnosis of HPP during the antenatal period. Prenatal diagnosis can be complicated by polyhydramnios, and the pregnancy would likely be considered high risk [2]. Procedures for high-risk pregnancies include amniocentesis, additional ultrasounds, genetic analysis, and possibly genetic counseling for the family.

Physical and occupational therapists have an important role in managing all forms of childhood HPP. In addition to providing intervention to improve bone and muscle strength, they also assess changes in gross motor, fine motor, and cognitive development through standardized tools. The Bayley-III should be used in patients up to 42 months of age [41], the Peabody Developmental Motor Scales, Second Edition, in patients from 43 to 71 months of age [42], and the Bruininks-Oseretsky Test of Motor Proficiency, Second Edition, in patients 72 months of age or older [43]. Therefore, adequate provision of therapists is important for improving outcomes in children with HPP.

The diverse spectrum of signs, symptoms, and complications of HPP resulted in resource utilization patterns that varied widely across the 3 patients in this case series. Such variation in healthcare requirements was particularly evident when the needs of Patient 1, whose manifestations were the most severe, were compared with those of Patients 2 and 3. As might be expected, Patient 1 used the greatest healthcare resources, primarily in the form of procedures related to respiratory support and exhaustive inpatient services such as those provided in the PICU. It is important to note that the 3 patients described in this article were selected as representative cases; however, every patient with HPP requires individualized treatment based on presentation and availability of local resources. Further, the resources available within the UK health system may differ from those available in other countries or regions or between local institutions and specialty centers such as those in this case series.

Asfotase alfa has been authorized by regulatory authorities in various parts of the world, including United States, Canada, Europe, Australia, and Japan [35–39]. The healthcare resource utilization for Patient 1 was based on survival with treatment with asfotase alfa. Recent clinical study data have shown that treatment with asfotase alfa improves musculoskeletal manifestations, growth, respiratory function, and motor function of infants and children with HPP [44, 45]. Thus, had Patients 2 and 3 been treated with asfotase alfa, we might have seen a reduction in healthcare resource utilization, with the exception of those resources associated with craniosynostosis, which to date has not shown improvement with asfotase alfa treatment. Nevertheless, patients receiving treatment with asfotase alfa require medical evaluation and treatment from variety of medical and allied healthcare professionals.

## Conclusions

In summary, we described healthcare resource utilization in 3 patients with HPP who demonstrated a spectrum of clinical manifestations. Data from this study may help in identifying and mobilizing resources for the management of HPP in multidisciplinary tertiary pediatric units.

## Abbreviations

ALP: Alkaline phosphatase
Bayley-III: Bayley Scales of Infant and Toddler Development, Third Edition
CPAP: Continuous positive airway pressure
CT: Computed tomography
DXA: Dual-energy x-ray absorptiometry
EEG: Electroencephalogram
HPP: Hypophosphatasia
MRI: Magnetic resonance imaging
NHS: National Health Service
NICU: Neonatal intensive care
PICU: Pediatric intensive care unit
PLP: Pyridoxal 5′-phosphate
PPi: Inorganic pyrophosphate
TNSALP: Tissue-nonspecific alkaline phosphatase

## References

[1] Weiss MJ, Cole DE, Ray K, Whyte MP, Lafferty MA, Mulivor RA (1988). A missense mutation in the human liver/bone/kidney alkaline phosphatase gene causing a lethal form of hypophosphatasia. Proc Natl Acad Sci U S A.

[2] Whyte MP. Hypophosphatasia and how alkaline phosphatase promotes mineralization. In: Thakker RV, Whyte MP, Eisman JA, Igarashi T, editors. Genetics of bone biology and skeletal disease. 2nd ed. San Diego, CA: Elsevier (Academic Press); 2018;481-504

[3] Rockman-Greenberg C (2013). Hypophosphatasia. Pediatr Endocrinol Rev.

[4] Hypophosphatasia FD (1957). Am J Med.

[5] Mornet E, Yvard A, Taillandier A, Fauvert D, Simon-Bouy B (2011). A molecular-based estimation of the prevalence of hypophosphatasia in the European population. Ann Hum Genet.

[6] Whyte MP (2017). Hypophosphatasia: an overview for 2017. Bone.

[7] Fleisch H, Russell RG, Straumann F (1966). Effect of pyrophosphate on hydroxyapatite and its implications in calcium homeostasis. Nature.

[8] Russell RG (1965). Excretion of inorganic pyrophosphate in hypophosphatasia. Lancet.

[9] Linglart A, Biosse-Duplan M (2016). Hypophosphatasia. Curr Osteoporos Rep.

[10] Baumgartner-Sigl S, Haberlandt E, Mumm S, Scholl-Burgi S, Sergi C, Ryan L (2007). Pyridoxine-responsive seizures as the first symptom of infantile hypophosphatasia caused by two novel missense mutations (c.677T>C, p.M226T; c.1112C>T, p.T371I) of the tissue-nonspecific alkaline phosphatase gene. Bone.

[11] Whyte MP, Mahuren JD, Vrabel LA, Coburn SP (1985). Markedly increased circulating pyridoxal-5′-phosphate levels in hypophosphatasia. Alkaline phosphatase acts in vitamin B6 metabolism. J Clin Invest.

[12] Barvencik F, Beil FT, Gebauer M, Busse B, Koehne T, Seitz S (2011). Skeletal mineralization defects in adult hypophosphatasia—a clinical and histological analysis. Osteoporos Int.

[13] Collmann H, Mornet E, Gattenlohner S, Beck C, Girschick H (2009). Neurosurgical aspects of childhood hypophosphatasia. Childs Nerv Syst.

[14] Berkseth KE, Tebben PJ, Drake MT, Hefferan TE, Jewison DE, Wermers RA (2013). Clinical spectrum of hypophosphatasia diagnosed in adults. Bone.

[15] Coe JD, Murphy WA, Whyte MP (1986). Management of femoral fractures and pseudofractures in adult hypophosphatasia. J Bone Joint Surg Am.

[16] Beck C, Morbach H, Wirth C, Beer M, Girschick HJ (2011). Whole-body MRI in the childhood form of hypophosphatasia. Rheumatol Int.

[17] Kozlowski K, Sutcliffe J, Barylak A, Harrington G, Kemperdick H, Nolte K (1976). Hypophosphatasia. Review of 24 cases. Pediatr Radiol.

[18] Moulin P, Vaysse F, Bieth E, Mornet E, Gennero I, Dalicieux-Laurencin S (2009). Hypophosphatasia may lead to bone fragility: don’t miss it. Eur J Pediatr.

[19] Silver MM, Vilos GA, Milne KJ (1988). Pulmonary hypoplasia in neonatal hypophosphatasia. Pediatr Pathol.

[20] Teber S, Sezer T, Kafali M, Kenderli T, Siklar Z, Berberoglu M (2008). Hypophosphatasia associated with pseudotumor cerebri and respiratory insufficiency. Indian J Pediatr.

[21] Balasubramaniam S, Bowling F, Carpenter K, Earl J, Chaitow J, Pitt J (2010). Perinatal hypophosphatasia presenting as neonatal epileptic encephalopathy with abnormal neurotransmitter metabolism secondary to reduced co-factor pyridoxal-5′-phosphate availability. J Inherit Metab Dis.

[22] Schmidt T, Mussawy H, Rolvien T, Hawellek T, Hubert J, Ruther W (2017). Clinical, radiographic and biochemical characteristics of adult hypophosphatasia. Osteoporos Int.

[23] Mohn A, De Leonibus C, de Giorgis T, Mornet E, Chiarelli F. Hypophosphatasia in a child with widened anterior fontanelle: lessons learned from late diagnosis and incorrect treatment. Acta Paediatr. 2011;100:e43–6. 10.1111/j.1651-2227.2011.02228.x 21342251

[24] Eade AW, Swannell AJ, Williamson N (1981). Pyrophosphate arthropathy in hypophosphatasia. Ann Rheum Dis.

[25] Seshia SS, Derbyshire G, Haworth JC, Hoogstraten J (1990). Myopathy with hypophosphatasia. Arch Dis Child.

[26] Chuck AJ, Pattrick MG, Hamilton E, Wilson R, Doherty M (1989). Crystal deposition in hypophosphatasia: a reappraisal. Ann Rheum Dis.

[27] Reibel A, Maniere MC, Clauss F, Droz D, Alembik Y, Mornet E (2009). Orodental phenotype and genotype findings in all subtypes of hypophosphatasia. Orphanet J Rare Dis.

[28] Whyte MP, Zhang F, Wenkert D, McAlister WH, Mack KE, Benigno MC (2015). Hypophosphatasia: validation and expansion of the clinical nosology for children from 25 years experience with 173 pediatric patients. Bone.

[29] Whyte MP (2016). Hypophosphatasia - aetiology, nosology, pathogenesis, diagnosis and treatment. Nat Rev Endocrinol.

[30] Weber TJ, Sawyer EK, Moseley S, Odrljin T, Kishnani PS (2016). Burden of disease in adult patients with hypophosphatasia: results from two patient-reported surveys. Metabolism.

[31] Lopez-Bastida J, Oliva-Moreno J (2010). Cost of illness and economic evaluation in rare diseases. Adv Exp Med Biol.

[32] Angelis A, Kanavos P, Lopez-Bastida J, Linertova R, Nicod E, Serrano-Aguilar P (2015). Social and economic costs and health-related quality of life in non-institutionalised patients with cystic fibrosis in the United Kingdom. BMC Health Serv Res.

[33] Bradley JM, Blume SW, Balp MM, Honeybourne D, Elborn JS (2013). Quality of life and healthcare utilisation in cystic fibrosis: a multicentre study. Eur Respir J.

[34] Cavazza M, Kodra Y, Armeni P, De Santis M, Lopez-Bastida J, Linertova R (2016). Social/economic costs and health-related quality of life in patients with Duchenne muscular dystrophy in Europe. Eur J Health Econ.

[35] Government of Canada. Summary Basis of Decision - Strensiq - Health Canada. October 13, 2016. https://hpr-rps.hres.ca/reg-content/summary-basis-decision-detailTwo.php?linkID=SBD00190. Accessed 15 Feb 2018.

[36] Strensiq [package insert]. Boston: Alexion Pharmaceuticals, Inc.; 2018.

[37] Strensiq [summary of product characteristics]. Rueil-Malmaison: Alexion Europe; 2017.

[38] Strensiq Australia [package insert]. Frenchs Forest NSW Australia: Alexion Pharmaceuticals Australasia; 2016.

[39] Strensiq [prescribing information]. Tokyo: Alexion Pharma GK; 2015.

[40] Whyte MP, Wenkert D, McAlister WH, Mughal MZ, Freemont AJ, Whitehouse R (2009). Chronic recurrent multifocal osteomyelitis mimicked in childhood hypophosphatasia. J Bone Miner Res.

[41] Bayley N (2006). Bayley scales of infant and toddler development. Administration Manual.

[42] Folio MR, Fewell RR (2000). Peabody developmental motor scales.

[43] Deitz JC, Kartin D, Kopp K (2007). Review of the Bruininks-Oseretsky test of motor proficiency, second edition (BOT-2). Phys Occup Ther Pediatr.

[44] Whyte MP, Greenberg CR, Salman NJ, Madson K, Mhanni A, Weber TJ (2012). Enzyme-replacement therapy in life-threatening hypophosphatasia. N Engl J Med.

[45] Whyte MP, Madson KL, Phillips D, Reeves A, McAlister WH, Yakimoski A (2016). Asfotase alfa therapy for children with hypophosphatasia. JCI Insight.

